# APSP Journal of Case Reports: Looking Backward - Looking Forward

**Published:** 2014-04-01

**Authors:** Bilal Mirza, Jamshed Akhtar

**Affiliations:** Department of Pediatric Surgery, The Children’s Hospital and the Institute of Child Health Lahore, Pakistan; Department of Pediatric Surgery, National Institute of Child Health Karachi, Pakistan

We are now in the 4th year of regular publication of APSP Journal of Case Reports (AJCR).Tracing our short journey we feel proud that many milestones have been attained that were entrusted upon editorial board by The Association of Paediatric Surgeons of Pakistan (APSP) in year 2010. The team worked untiringly and also tried to be innovative. It used latest technologies available with limited resources both in terms of human resource and hardware. Considering the uniqueness of our specialty and importance of cases, it was kept up-front to encourage researchers to contribute new knowledge to the world. Case report format thus suited well to our goals. The first issue was available online on 14th August 2010[1]. Two issues were published in the first year, followed by three issues in the subsequent volumes. At present AJCR has published 11 issues. The AJCR was indexed with PubMed and PubMed Central in September 2012.

Reviewing the submission trends to the AJCR, we retrieved the record of all the manuscripts published till date and analysed the data with Epiinfo version 7. The results are shown in figures (Fig. 1,2) and tables (Table 1,2).

**Figure:**
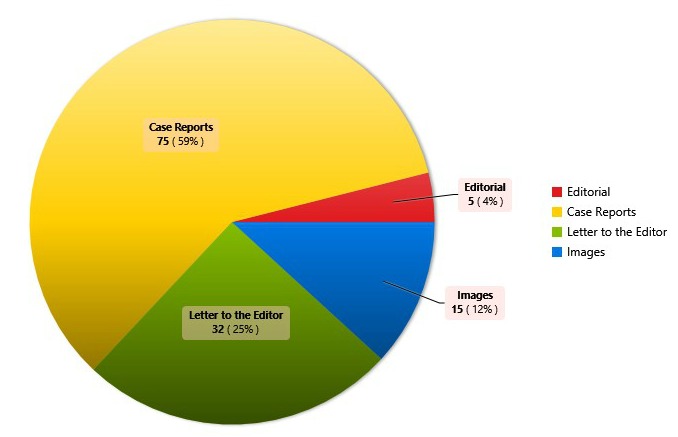
Figure 1:Types of manuscripts published

**Figure:**
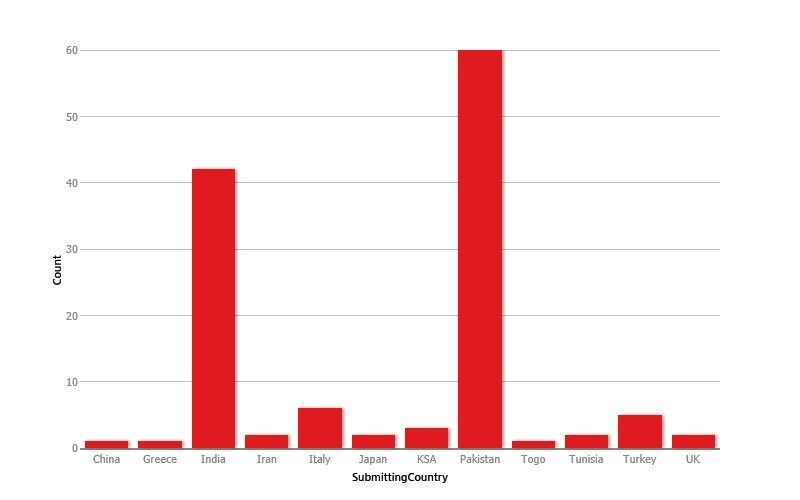
Figure 2:Submission trend with respect to country of origin.

**Figure:**
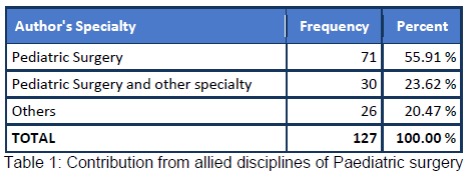
Table 1:Contribution from allied disciplines of Paediatric surgery

**Figure:**
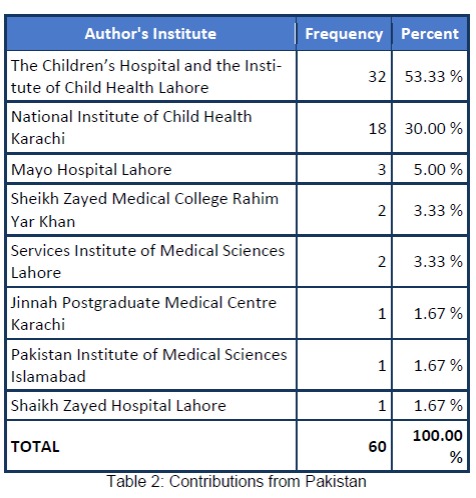
Table 2:Contributions from Pakistan

The analysis depicted that the majority of the manuscripts were in the category of case reports. AJCR received submissions from various pediatric surgery and non-pediatric surgery units of Pakistan and various other countries. Major contribution to AJCR was from Sub-continent; however, only few pediatric surgery units of Pakistan made the contribution. Indexing with world renowned database like PubMed and PubMed Central [2] resulted in wide visibility of AJCR and a rise in submissions from many countries including from Europe is now apparent. Various articles of AJCR are being cited in the literature. We expect to have an official Impact Factor in the coming years. We hope to take AJCR to new heights in collaboration with our contributors.

## Footnotes

**Source of Support:** Nil

**Conflict of Interest:** None declared

